# Preparedness of civil society in Botswana to advance disability inclusion in programmes addressing gender-based and other forms of violence against women and girls with disabilities

**DOI:** 10.4102/ajod.v9i0.664

**Published:** 2020-07-28

**Authors:** Jill Hanass-Hancock, Nomfundo Mthethwa, Malebogo Molefhe, Tshiamo Keakabetse

**Affiliations:** 1 Gender and Health Research Unit, South African Medical Research Council (SAMRC), Durban, South Africa; 2 School of Health Science, University of KwaZulu-Natal, Durban, South Africa; 3 Private, Gaborone, Botswana; 4 Institute for Development and Management, Gaborone, Botswana

**Keywords:** gender-based violence (GBV), violence, HIV, Botswana, participation

## Abstract

**Background:**

In low-income and middle-income countries women and girls with disabilities are more likely to experience violence than those without disabilities. Non-governmental organisations (NGOs) and disabled people’s organisations (DPOs) can help to address this. However, in countries like Botswana we know little about the preparedness of NGOs and DPOs to increase inclusion in and access to programmes addressing violence.

**Objectives:**

To explore the capacity and preparedness of NGOs and DPOs to ensure that women and girls with disabilities can participate in and access programmes addressing violence.

**Methods:**

A qualitative study was undertaken using interviews with 17 NGOs and DPOs in Botswana to understand the organisations’ level of and ability to deliver programmes addressing violence against women and girls.

**Results:**

Both NGOs and DPOs lack elements of universal design and reasonable accommodation, and thus are inaccessible to some people with disabilities. Some programmes address violence against women but lack skills and resources to accommodate people with disabilities. In contrast, DPOs work with people with disabilities, but lack focus on violence against women with disabilities. Participants identified opportunities to fill these gaps, including adaptation of policies and structural changes, training, approaches to mainstream disability across programmes, development of disability-specific interventions and improved networking.

**Conclusions:**

Botswana’s NGOs and DPOs are well positioned to address violence against women and girls with disabilities, but need to increase their accessibility, staff knowledge and skills and disability inclusion. Training, resource allocation and participation of women with disabilities in NGOs and DPOs is needed to drive this change.

## Introduction

Violence against women is a serious violation of women’s rights and a public health concern (World Health Organization 2013). Globally, violence against women and girls is also an issue of scale: 35% of women have reported experiencing sexual or physical violence from a partner or non-partner worldwide (World Health Organization 2013). Women and girls with disabilities are even more vulnerable to all forms of violence, including physical, emotional, economic, structural and sexual violence, compared to men and women without disabilities (Dunkle et al. 2018; Hughes et al. 2012; Jones et al. 2012; UNWomen 2013). Literature shows that this vulnerability is fuelled by double discrimination based on disability and its intersection with negative gendered norms and attitudes (Kvam 2004, 2005; Kvam & Braathen 2006, 2008; UNWomen 2013). In societies where gender inequality and violence against women are endemic, women and girls with disabilities are therefore even more likely to experience violence (UNWomen 2013; USAID Botswana 2014).

Internationally data on prevalence of violence against women and girls with disabilities are sparse for middle-income and low-income countries (Hughes et al. 2012; Jones et al. 2012). Recently, the ‘What Works’ programme has released data on intimate-partner violence (IPV) revealing that women with disabilities are two to four times more likely to experience IPV than their peers without disabilities (Dunkle et al. 2018). Within the eastern and southern African region a body of research is also emerging showing that women and girls with disabilities are not only more vulnerable to all forms of violence (including intimate-partner and gender-based violence), but that they are also more likely to experience confounding risk factors of violence such as multidimensional poverty (Banda 2005; Eide 2003; Eide & Kamaleri 2009; Eide, Khupe & Mannan 2014; Eide & Loeb 2006; Hanass-Hancock 2015; Mitra, Posarac & Vick 2013; South African Department of Social Development 2016), inequality and discrimination based on gender and disability (Charowa 2005; Dunkle et al. 2018; Kvam & Braathen 2008; Zimbabwe Parents of Handicapped Children Association, unknown), poor access to services including sexual and reproductive health rights (SRHR) and sexuality education programmes (UNAIDS 2014; UNFPA 2018a, 2018c), and increased likelihood of violence in childhood (Kvam & Braathen 2006; Save the Children 2011).

Botswana is a middle-income country with high levels of violence against women and girls. The 2018 Relationship Study indicates that 37% of women in Botswana have experienced some form of violence in their lifetime, including both partner and non-partner violence (Botswana Ministry of Nationality Immigration and Gender Affairs 2018). The same study reveals that 30% of women experienced violence in the last year. The most commonly reported form of violence was emotional IPV, followed by physical, sexual and economic IPV. Child sexual abuse is also reported as a significant risk factor for exposure to violence in adulthood. Both the 2012 gender-based violence (GBV) indicator report and the 2018 Relationship Study reveal that exposure to violence leads to physical injuries, sexual and reproductive health issues (sexually transmitted infections, HIV) and poor mental health among women (Botswana Ministry of Nationality Immigration and Gender Affairs 2018; Machisa & Van Dorp 2012). Hence, the level of violence against women and girls is alarmingly high in Botswana and this has a wide-ranging impact on individuals, families, communities and the country as a whole (Bloom & Curran 2015; USAID Botswana 2014). The country is therefore specifically focusing on prevention of violence (Botswana Ministry of National Immigration and Gender Affairs 2016; Hanass-Hancock et al. 2018b).

Information on violence against women and girls with disabilities is sparse in Botswana. For instance, the country’s 2012 GBV Indicator Study (Machisa & Van Dorp 2012) did not include information on people with disabilities. The 2018 Relationship Study (new GBV indicator survey) included for the first time a disability indicator revealing that people with disabilities experience high levels of partner and non-partner violence. The study also shows that women with disabilities are more likely to experience violence than men with disabilities (Botswana Ministry of Nationality Immigration and Gender Affairs 2018). Apart from this information we have very little understanding of the levels and types of violence women with disabilities experience in the country, what risk factors increase their vulnerability and what interventions can reduce these risks.

The first data describing the risk factors of violence against women and girls with disabilities became available through the ALIGHT Botswana project. The associated qualitative study revealed that women with disabilities experience all forms of violence, but that emotional and sexual violence are of particular concern (Hanass-Hancock et al. 2018a). The study also showed that the context perpetuating violence against this group is shaped by: harmful individual attitudes and lack of knowledge on the part of survivors, perpetrators and service providers, negative sociocultural norms related to disability and gender, disability-related barriers in the environment, lack of access to resources and a lack of disability inclusion in the country’s SRHR and GBV policies and strategic plans. In addition, the project’s inception phase suggested that inclusion of women and girls with disabilities was absent from most non-governmental organisations (NGOs) working in the context of violence (including those focusing on GBV, SRHR and HIV), while disabled people’s organisations (DPOs) lacked focus on violence against women and girls with disabilities (Hanass-Hancock et al. 2018b).

Nevertheless, NGOs and DPOs are often believed to fill service gaps, sensitise communities and reach out to marginalised populations in low-income and middle-income countries (Dunkle et al. 2018). For instance, NGOs have contributed significantly to the increase in service delivery and outreach of strategic programmes, such as those for HIV and AIDS in Africa (Handicap International 2014). Similarly, DPOs are seen as important partners for disability rights programming, while disability service organisations have been a cornerstone to reach people with disabilities in resource-poor settings (e.g. Humanity and Inclusion, Christopher Blind Mission, Sight Savers, Leonard Cheshire etc.) (Handicap International 2013, 2014; UNAIDS 2017). International programmes focusing on violence in low-income and middle-income countries such as the ‘What Works’ and the ‘Make It Work’ programmes recommend partnering with DPOs (Dunkle et al. 2018; Handicap International 2013). These programmes claim that DPO and NGO involvement is an ‘effective strategy’ to ensure inclusion of women and girls with disabilities in mainstream violence prevention programmes (Dunkle et al. 2018). Currently we have little evidence of the capacity or preparedness of NGOs and DPOs in resource-poor settings to ensure that women and girls with disabilities can access and benefit from programmes that are designed to prevent violence against women or girls in general (Hanass-Hancock et al. 2018b; UNFPA 2018a).

This article is part of the ALIGHT Botswana project, which focuses on increasing participation of women and girls with disabilities in programmes that address violence, SRHR or HIV in Botswana. The sub-study presented here focuses on the capacity and preparedness of NGOs and DPOs to ensure that women and girls with disabilities can participate in and access programmes addressing violence. It also identifies the gaps and opportunities for these NGOs and DPOs to drive inclusion of women and girls with disabilities in programmes addressing violence.

## Methods

The presented sub-study is imbedded in the larger ALIGHT Botswana study, which has four key objectives: focusing on identifying risk factors of violence against women and girls with disabilities, describing the experience of violence among women with disabilities, identifying the gaps and opportunities for NGOs and DPOs to increase access and participation and appraising the inclusiveness of SRHR, HIV and GBV policies and programmes (SAMRC, IDM & BCD 2017). All four sub-studies informed the training and capacity building of NGOs and DPOs as well as the parallel efforts to develop a disability policy and strategy in Botswana.

### Study design

The ALIGHT study used a participatory action learning and action research approach (PALAR) (Kearney & Zuber-Skerritt 2012; Wood & Zuber-Skerritt 2013; Zuber-Skerritt 2015). The PALAR approach integrates the concept of action learning with action research. It also integrates participatory elements and aims at positive social change for a just and better world for all human beings. The researchers in this study made specific efforts to ensure (1) participation and leadership of women and girls with disabilities, (2) participatory engagement of researchers with the target group and (3) mutual learning and actions towards greater inclusion of women and girls with disabilities in existing programmes.

This meant that the study team (researchers, fieldworkers, transcribers, facilitators) included women with disabilities, enabled leadership of women with disabilities and translated research results into actions in the partnering DPOs and NGOs (in the form of training and strategy development).

### Sampling

The Botswana Council for the Disabled (BCD) functioned as a project partner and gatekeeper for the fieldwork. The BCD is an umbrella body for all DPOs in Botswana and has collaborative links to NGOs working in the fields of GBV, HIV and SRHR. Through this network and a collaborative inception phase the ALIGHT project officer identified and recruited potential participants for key informant interviews (KII). Participants for the sub-study with NGOs and DPOs had to be senior managers or directors of the respective organisations in three purposely selected areas (areas that had head offices of the major DPOs and NGOs working in the country).

### Research tools

**FIGURE 1:**
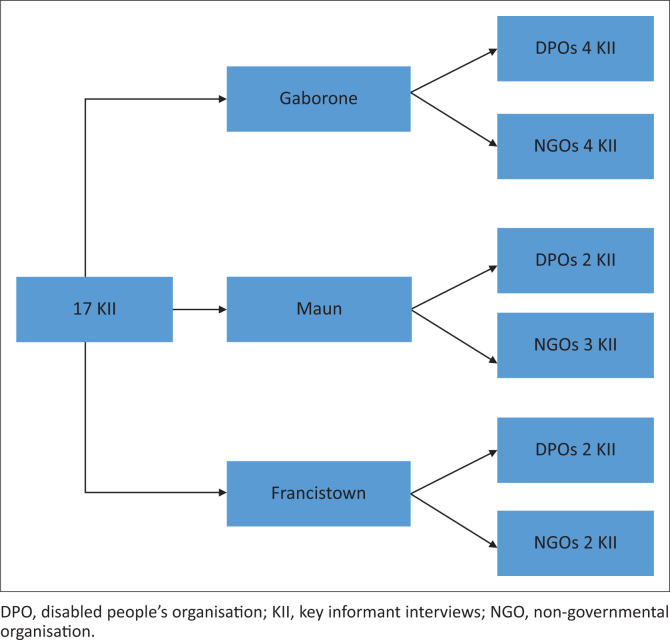
Sampling framework for the key informant interviews.

We conducted 17 KIIs with NGOs and DPOs in Gaborone, Maun and Francistown (Figure 1). These KIIs were guided by a qualitative question guide and a disability inclusion audit in the form of a self-designed checklist. Both tools were developed using the Convention on the Rights of Persons with Disabilities (CRPD) and the Action Linking Initiatives on Violence Against Women and HIV Everywhere (ALIV[H]E) framework as guiding frameworks to understand the organisations’ context, level of disability inclusion and ability to deliver programmes addressing violence against women and girls including those with disabilities (Salamanda Trust et al. 2017).

The interview guide prompted information related to the organisational profile, the organisations’ services and activities, the level of inclusion of people with disabilities, perceptions of gaps, opportunities and potential strategic actions to increase inclusion and participation.

The checklist prompted the participants’ perceptions in six domains important for disability inclusion and accessibility of NGOs and DPOs: level of disability accessibility through universal design and reasonable accommodation, disability-related sensitisation and training of staff, the ability to screen and identify disability, the ability to provide or refer to disability services, linkages to poverty alleviation programmes focusing on people with disabilities and linkages to civil society, including those focusing on violence. All six domains were prompted through several questions, scored and then reported as frequencies for the group of NGOs and DPOs separately (see Table 2 and Table 3). The checklist cannot be seen as a comprehensive tool but is rather a first snapshot prompting some basic elements that need to be considered when trying to include people, particularly women and girls with disabilities, in programmes addressing violence. Hence the checklist was used as a guiding tool to systematically collect data and has been inspired by similar checklists used to assess inclusion in healthcare services providing services on HIV, SRHR and violence (Hanass-Hancock & Alli 2015). A separate article will focus on this tool.

The data from the KIIs were transcribed, translated and analysed using guided content analysis. A team of three researchers developed a case study report for each of the 17 NGOs and DPOs. These case studies included descriptions of four main themes: the organisational profile, including levels of disability inclusion (universal design, reasonable accommodation, disability identification and referral), the organisational capacity to address violence against women, including those with disabilities, perceptions of internal and external opportunities to improve participation and inclusion and perceptions of important roles and involvement of key stakeholders.

Thereafter we compared and synthesised the 17 case studies for emerging similarities and differences across all organisations. These in-depth descriptions were discussed by the research team, paying specific attention to variations across all three selected geographical areas, between NGOs and DPOs and across different disability types.

### Ethical considerations

Information about the study was provided in writing and discussed with the participants verbally before the interviews. The information provided explained the purpose of the study, procedures, potential risks and benefits, contact information and voluntary nature of the interviews. Prior to the interview an informed consent form was signed. The study was approved by the South African Medical Research Council (SAMRC EC019-10/2017) and the Botswana Ministry of Health and Wellness (DPDME 13/18/1) and endorsed by the Botswana Office of the President.

## Results

Representatives from eight NGOs and nine DPOs participated in the KIIs (see Table 1). The NGOs’ work focused on GBV, violence against women and girls, roles of men and boys, legal services or implementation of HIV programmes. Their core activities included advocacy, community engagement and service delivery, meaning they were working on addressing gender inequality, negative attitudes and discrimination. All of the NGOs had central offices with some of them supporting branches in several provinces.

**TABLE 1:** Sampling for key informant interviews.

Variables	Gaborone (*N* = 9)		Francistown (*N* = 4)		Maun (*N* = 4)	
	*n*	%	*n*	%	*n*	%
Disabled people’s organisation representatives	5	56.0	2	50	2	50
Non-governmental organisation representatives	4	44.0	2	50	2	50
Women	5	55.5	2	50	0	0
People with disabilities	2	22.2	0	0	1	25
Caregiver of children with disabilities	1	11.0	0	0	0	0

The DPOs included organisations self-representing people with disabilities as well as disability service organisations. With the exception of three organisations, the DPOs focused on one particular disability type only (e.g. people with hearing, visual, intellectual or physical impairments). All DPOs had only one headquarters office and some were operating from homes as they were situated at grassroots level with few resources. The DPOs focused mainly on advocacy and networking. Some also provided support groups or specialised services (e.g. sign language interpretation, Braille, rehabilitation services) or else they had focused programmes (e.g. accessibility to sport activities or income-generating activities). One disability service organisation had previously conducted a project focusing on violence and two DPOs had been involved in HIV projects. Only three organisations had people with disabilities in leadership positions, although none was a woman with a disability (Table 1). While DPOs employed people with disabilities, none of the NGOs employed people with disabilities.

The results from the KIIs relate to four main themes: the organisational profile and level of disability inclusion, the organisational capacity to address violence against women and girls with disabilities, perceptions of internal and external opportunities to improve participation/inclusion and perceptions of roles and involvement of key stakeholder.

### The organisational profile and level of disability inclusion

The managers and directors of the participating organisations provided information related to the level of inclusion in the organisations’ policies and strategic plans, the accessibility of activities and facilities, the training and capacity of staff and the linkages to other NGOs and DPOs, disability services, violence and poverty alleviation programmes.

Participants from both NGOs and DPOs held the notion that their organisation’s policies and strategic approaches included people with disabilities (Table 2). In the interviews NGO representatives shared that ‘their services are for all people’, with some believing that this general statement is enough to include people with disabilities on a policy and programme level. In addition, two NGO participants shared that they believed that they ‘shouldn’t know the disability of people’ and should treat all people equally.

‘We have a core belief [*that*] one shouldn’t know the disabilities of people, some might have a learning disability, some might have physical disability that can be seen, but we offer our services to the public for women and men.’ (Manager of NGO working with men and boys)

**TABLE 2:** Self-reported assessment using disability inclusion checklist.

Items prompted	NGOs (*n* = 8)	DPOs (*n* = 9)
***Perception of organisational policies and programmes***		
Identify disability indicators for monitoring and evaluation	2	5
Identify that people with disabilities are at risk of violence	6	7
Identify collaboration with DPOs	7	7
Identify barriers to access the programme’s services	5	7
Identify measures to overcome disability-related access barriers	6	7
Mainstream disability into its programmatic areas and objectives	4	8
Provide targeted interventions for people with disabilities	4	8
***Perception of accessibility of buildings and facilities***		
Include ramps to all buildings (or are all on one level)	0	2
Include crucial services on the ground floors (or lifts)	1	6
Include doors wide enough to fit a wheelchair	5	5
Include wheelchair accessible toilet (wide enough doors, space and railings)	1	2
Include directions on key areas in Braille (e.g. lifts, signposts)	1	0
***Perception of disability accommodation***		
Include provision of disability or accessibility desk or focal person in organisation	1	4
Include provision of furniture to accommodate physical disabilities through height adjustments, etc.	0	1
Include provision of sign language interpretation when needed	0	1
Include information in Braille or in audio format	1	1
Include simplified information for people with intellectual disabilities (e.g. pictures)	2	2
***Perceptions of training of staff***		
Provided anti-stigma training focusing on disability	3	5
Included training on sign language interpretation and Braille	1	4
Included training on the interrelationship of disability and gender-based violence (sensitisation)	5	4
Included training to screen for disability including mental health in general services such as antiretroviral therapy	4	3
Included information on referral services such as educational support, community-based rehabilitation	6	5
***Perception of linkage to poverty alleviation programmes***		
Links to employment programmes that cater for people with disability	4	6
Links to food security programmes that include people with disabilities	3	5
Links to sheltered employment for people with disabilities	1	2
Includes referral system to social work, grants or business loans	6	5
***Perception of established referral system***		
Has screening tools to identify disability including mental health problems available	2	3
Can refer from gender-based violence programme or service to disability specific services	6	8
Can refer from gender-based violence programme to judicial services that can support people with disabilities	7	8
Can refer to peer support, e.g. DPOs or NGOs targeting people with disabilities	7	8
***Connectivity to civil society and social services***		
Connected to a women crisis centres	6	5
Connected to community-based rehabilitation	7	8
Connected to food security programmes	6	6
Connected to DPOs	5	6
Connected to disability service organisations	8	9
Connected to local police	7	9
Connected to local social workers	8	9
Connected to traditional authorities	8	9

DPO, disabled people’s organisation; NGO, non-governmental organisation.

Criticising this practice of ‘inclusion via default’, one NGO representative emphasised the importance of prioritising disability in the strategies and policies of NGOs. The participant highlighted that without prioritisation resources were difficult to allocate to disability needs. The participant also explained that awareness around disability was only emerging and that therefore NGO strategies and programmes did not yet include or prioritise disability. The lack of inclusion and prioritisation in the strategies and policies of NGOs and funding agencies leads to lack of resources and skills that are needed to accommodate people with disabilities in the work of NGOs.

‘Disability is an emerging issue … and we need someone who has those skills as part of our team … however because it [*disability*] is not a strategic area we can’t give it priority. [*As a consequence*] even when we are talking with regards to resources we … need to demonstrate that there are women with disabilities out there who require our services.’ (Manager of NGO focusing on violence against women)

The DPO representatives believed their policies or strategic plans were disability inclusive because they were a DPO and informed about disability issues by their constituency. The DPOs usually catered for the disability type they focused on, and employed people with disabilities (while NGOs did not employ people with disabilities). However, none of the DPOs was familiar with the concepts of universal design and reasonable accommodation and spoke more generally about accessibility and inclusion.

‘Our services … are open to any person with visual impairment regardless if she or he is our member or not as long as he or she is visually impaired. … The challenge is access, how to reach them because most of them live in villages while we are limited by resources to go to the villages. Access to information, they [*people with disabilities*] don’t have access to information.’ (Representative of DPO for people with visual impairments)

When prompted with the disability inclusion checklist, 15 out of the 17 organisations lacked basic measures of universal design or reasonable accommodation or both (Table 2). For instance, none of the NGOs and only two of the DPOs included ramps to their buildings. Only one NGO and two DPOs had wheelchair accessible toilets and only one organisation included signs in Braille. Similarly, very few organisations provided sign language, Braille or simplified information (Table 2). Some of the NGOs specifically criticised this in their interviews.

‘They [*the NGO’s facilities*] do not accommodate, it does not even allow them [*people with disabilities*] to come forth and access services especially those ones using the wheelchair.’ (Manager of NGO focusing on HIV prevention)

The DPOs tended to provide accommodation for the disability type they focused on, while only very few NGOs included some measures of universal design or reasonable accommodation. Only two disability service organisations provided a more comprehensive set of universal design and reasonable accommodation measures across disability types. As a result, DPOs and NGOs were mostly inaccessible. The researchers, who included women with disabilities, therefore found it very difficult to conduct the fieldwork in some settings and alternative arrangements had to be made.

Furthermore, the KIIs and disability inclusion checklist revealed that both NGOs and DPOs had undergone staff training, with NGOs having more training on violence and HIV-focused organisations and DPOs more training related to disability. Most organisations lacked training on the intersection of disability and violence. All NGOs and DPOs indicated that they had established referral links with other organisations or services, hence referrals between DPOs, disability services and NGOs focusing on violence and HIV was possible. However, in most organisations staff were not trained to identify disability or use screening tools, hence less visible disabilities are more likely to be overlooked.

In addition, all representatives believed that their organisation was well connected to other civil society organisations. Generally, NGOs provided better links to income-generating opportunities or poverty alleviation programmes than DPOs. However, very few DPOs or NGOs made specific efforts to provide access to economic resources for women with disabilities.

### Organisational capacity to address violence against women and girls with disabilities

The NGOs and DPOs had very different profiles in terms of their ability to deliver services to women with disabilities and increase their participation in programmes addressing violence or related issues (e.g. HIV or SRHR). Most NGOs had established programmes focusing on GBV, HIV or SRHR, but lacked inclusion of people with disabilities, while DPOs were better established to reach and support people with disabilities, but lacked resources to provide services related to GBV, HIV and SRHR.

Participants from NGOs revealed that they had several offices and outreach activities in the country and were therefore able to reach women across districts.

‘We are in about sixteen districts countrywide and this is an opportunity to enhance the participation of women with disabilities. … We can mobilise the communities and ensure that they know stigmatising people with disabilities is not a good thing.’ (Manager of NGO focusing on HIV prevention)

The NGO representatives also explained that they provided a number of services including provision of information on GBV or HIV, counselling, testing and community engagement and sensitisation. They reached out to communities and services (e.g. schools, police and clinics) and engaged with traditional authorities and government.

‘The organisation deals with GBV primarily by giving information especially to girls and women and we recently had a project … where we were giving children in school and in the community messages on gender-based violence.’ (Manager of NGO focusing on violence against women)

However, representatives from NGOs highlighted that their facilities were physically inaccessible and that their staff lacked skills and competence to accommodate people with disabilities. Hence they emphasised that they needed training and better linkage to disability service providers.

‘You need people who have the competence to work with people with different disabilities, for example … the feedback that we get from the Deaf and the Blind is that we have counsellors who are not trained and expect them to give them services, how are they (the councillors) going to be working if they have not been trained. … We need to look into that.’ (Manager of NGO focusing on HIV prevention)

One NGO member of an AIDS service organisation identified the lack of skills and disability accommodation as the ‘weakness’ of NGOs’ programming, and linked it to ‘generalising people living with HIV’ while failing to address the diversity and different needs of their clients. This participant revealed that ignorance about disability leads to NGO services ‘being discriminative of people with disabilities’.

‘We do not have special services for people with disabilities and this is one of our weaknesses when it comes to programming. We generalise all people living with HIV; no emphasis on people with disabilities. … So you will find that our services are discriminative of them. … When there is a hearing impaired individual who has come for health services, there is no provision of sign language meaning that he or she is not catered for. But we assume that we are providing services to people infected and affected [*by HIV*]. It’s high time we mainstream disability issues in our programmes.’ (Manager of AIDS service organisation)

Capacity building was seen as key to improving service delivery for people with disabilities. The respondents identified the need for specialised services to enhance communication such as sign language interpretation or Braille but not the need to understand disability rights or services for other disability types such as intellectual disabilities. Hence, respondents were more aware of the communication needs of specific disability groups.

‘Capacity building. Let there be education and training. Let’s have people in services who can provide sign language where people with disabilities are free to access service without wondering who is going to help them. … I think education and training; if capacity building component includes issues like sign language and Braille, we would be doing much better.’ (Manager of NGO focusing on HIV prevention)

The NGO representatives also highlighted the need to better integrate violence interventions and HIV services, a trend that is currently also supported by the integrated sexual and reproductive health strategy of Botswana (Department of HIV Prevention and Care 2016). Integration was seen as important as the two topics were understood to overlap: people living with HIV may also have experienced violence (and vice versa), hence counsellors dealing with HIV had clients who also experienced violence and discrimination.

‘Integration. We have HIV testing projects but I know the issues of gender-based violence have not been included, but I feel that our counsellors could be given some training in gender-based violence cause they also deal with communities … given their exposure to issues of gender based violence, integration is very important in our days.’ (Manager of NGO focusing on HIV)

Participants from DPOs revealed that their organisations provided a network, support groups, opportunities for advocacy of specialised services (sign language, Braille) to their constituency. With the exception of one organisation, all DPOs had only one office, hence their reach was very local and restricted to the limited funding they had. As a result DPOs may be able to provide or refer to specialised services but have only localised reach to people with disabilities.

Representatives from DPOs also revealed that support and information in terms of violence and HIV was not provided by them through formal programmes but more informally through responding to emerging discussions, cases or request for legal support.

‘When they [*people with disabilities*] are … exposed to violence … our association … told them not to keep quiet, if they were not able to shout … immediately after the incident they should report.’ (Manager of DPO for people with intellectual disabilities)

Two DPO representatives revealed that their organisations had started to work in the context of violence and disability. For instance, one participant explained that their organisation ‘bridges the communication gap’ for deaf people by providing sign language interpretation and linking deaf people to suitable legal aid. The narrative below describes how DPOs can assist in the process of gaining access to justice and services:

Wherever there’s a problem identified, the officers [*the DPO staff*] will help the family or the woman or young girl to get help on any issue that they may have encountered. For example, we had one young deaf lady … who was sexually abused by another deaf individual, the officers helped the young girl to report the case to the police and also helped the young girl to get a fair hearing through enabling communication between the police and the victim … to ensure that women and deaf girls have fair representation. … We bridge the communication gap for them and enable access to services such as the police. If it is a legal issue we help to contact possible service providers, like Legal Aid Botswana, Department of Law, Botswana Independent Law Society, where they can get free assistance in terms of representation when they need to go to the court. (Manager of DPO for the Deaf)

A second participant revealed that their disability service organisation had one project that focused on disability rights which included discussions around violence and people with disabilities. In their engagement with the community on this topic the DPO identified barriers to accessing services as well as knowledge gaps about rights among people with disabilities.

‘We were doing a project in …, where we were focusing on girls and women with disabilities. We were looking at their rights and if they know their rights. We were also teaching them different types of rights, how they can access services and where they can report violence. In our fieldwork we realised that some people don’t know that certain violations are violence.’ (Manager of disability service organisation)

The DPO representatives also identified a number of challenges to reach out to people with disabilities. This included the DPOs’ lack of human and financial resources, capacity and their limited geographical reach.

‘The challenge is that we are a small grass roots DPO, we don’t have funds to travel, and we need resources to travel or maybe if we have identified someone’s needs, and we need to help, we are unable to help but we are able to advocate, we go to the relevant personnel to assist though it takes time due to financial resources.’ (Manager of DPO for physical and sensory disabilities)

Lack of resources was seen as linked not only to the limited outreach to people with disabilities but also to a lack of systematic work to identify and prevent violence and abuse. One DPO representative explained that their limited reach resulted in them only being able to respond to reported cases and not through outreach activities of the DPO.

‘The most hindrance from achieving our mandate is that of lack of resources, if we had sufficient resources we could engage more on the 16 days of activism activities to sensitize the nation about GBV. … Unfortunately as an association we only act on what has been reported to us about disabled people. We have not directly been involved as we don’t have a programme on GBV that caters for disabled people but somehow there are cases whereby we have been involved where women with disabilities were abused.’ (Manager of DPO for people with visual impairments)

Some DPO members discussed concrete ideas on how they could assist people with disabilities and what services they wanted to provide. This included income-generating activities, networking opportunities and advocacy around accessibility. One DPO member explained that they needed to discuss their needs for support with government.

‘We are challenged financially as an association, but our intention is to meet with government officials such as district commissioner. … We want to help in assisting disabled people with funds to start small businesses like gardens, bags, leather produce etc. at their homes and to liaise with government officials for any opportunities that can improve their well-being by also providing market places where they can show case their different crafts and sell their products in order to earn a living. There is also a need to ensure access to infrastructure developments such that wheelchair users can have full access. I was able to advocate for some government offices which offered direct services to disabled people such as Omang offices to be moved to ground floor.’ (Manager of DPO for people with physical disabilities)

Another DPO representative reported that they discussed issues with government representatives; however, providers of government initiatives and services were not always inclusive of people with disabilities and this provided barriers to DPOs to reach into the communities.

‘Firstly, we don’t have transport to bring us here [*people with physical disability*]. Secondly, when I ask for inclusion in socio-economic activities like Ipelegeng [*transport service*] and Tirelo Sechaba [*government initiative*], these service providers are reluctant to come and discuss issues we face as people with disabilities.’ (Manager of DPO for physical disabilities)

### Organisational opportunities to increase participation

Both NGOs and DPOs identified a number of internal change opportunities. These included employment and participation of people (particularly women) with disabilities, increasing accessibility (universal design and reasonable accommodation), training and capacity building and enhancing networking (Table 3).

**TABLE 3:** Identified organisational opportunities for change.

Items prompted	NGOs (*n* = 8)	DPOs (*n* = 9)
***Opportunities to adapt organisational policies and programmes***		
Identify disability indicators for monitoring and evaluation	2	5
Identify that people with disabilities are at risk of violence	7	7
Identify collaboration with DPOs	8	8
Identify barriers to access the programme’s services	5	7
Identify measures to overcome disability-related access barriers	7	7
Mainstream disability into its programmatic areas and objectives	5	8
Provide targeted interventions for people with disabilities	4	8
***Opportunities to increase accessibility of buildings and facilities***		
Include ramps to all buildings (or are all on one level)	4	6
Include crucial services on the ground floors (or lifts)	1	5
Include doors wide enough to fit a wheelchair	6	6
Include wheelchair accessible toilet (wide enough doors, space and railings)	2	5
Include directions on key areas in Braille (e.g. lifts, signposts)	1	3
***Opportunities to adapt disability accommodation***		
Include provision of disability or accessibility desk or focal person in organisation	2	6
Include provision of furniture to accommodate physical disabilities through height adjustments, etc.	0	5
Include provision of sign language interpretation when needed	1	6
Include information in Braille or in audio format	2	4
Include simplified information for people with intellectual disabilities (e.g. pictures)	3	3
***Opportunities to adapt training of staff***		
Provided anti-stigma training focusing on disability	3	6
Included training on sign language interpretation and Braille	2	6
Included training on the interrelationship of disability and gender-based violence (sensitisation)	5	7
Included training to screen for disability including mental health in general services such as antiretroviral therapy	3	6
Included information on referral services such as educational support, community-based rehabilitation	6	7
***Opportunities to adapt linkage to poverty alleviation programmes***		
Links to employment programmes that cater for people with disability	5	3
Links to food security programmes that include people with disabilities	7	8
Links to sheltered employment for people with disabilities	7	8
Includes referral system to social work, grants or business loans	8	8
***Opportunities to adapt established referral system***		
Has screening tools to identify disability including mental health problems available	7	6
Can refer from gender-based violence programme or service to disability specific services	7	8
Can refer from gender-based violence programme to judicial services that can support people with disabilities	6	7
Can refer to peer support, e.g. DPOs or NGOs targeting people with disabilities	8	9
***Opportunities to adapt to increase networking with civil society and social services***		
Connected to a women crisis centres	7	6
Connected to community-based rehabilitation	7	8
Connected to food security programmes	6	7
Connected to DPOs	8	9
Connected to disability service organisations	8	9
Connected to local police	8	8
Connected to local social workers	8	8
Connected to traditional authorities	8	8

DPO, disabled people’s organisation; NGO, non-governmental organisation.

In the interviews one manager of an NGO focused on HIV stated that although their organisation had existed for 21 years they had ‘never recruited or employed a woman with disability’ and as the organisation ‘is also an employer they have the opportunity to bring them [*women with disabilities*] on board’.

‘We can also improve our services by employing someone with disabilities in our organisation. We should also act synonymous to other stores such as SPAR who support disabled people by involving them through employment.’ (Manager from AIDS service organisation)

Furthermore, increased involvement of people with disabilities in capacity building programmes and the design of programmes were seen as important opportunities for change. Inclusion was also seen as a feasible strategy that could be sustained over time in the communities (rather than isolated projects).

‘The strategy that can be introduced is to involve the beneficiary in the learning programme because sometimes we come up with the programme as we feel, but people … have not been part of it from the beginning, so inclusion of beneficiaries to hear their thoughts, their challenges in the localities, I think that strategy could be sustained along within the communities.’ (Manager of AIDS service organisation)

Representatives from NGOs and DPOs also highlighted that disability sensitisation and training of staff to provide accommodation for disability was needed in their organisations. Accommodation measures needed for the Blind and Deaf were often highlighted, but not measures for those with intellectual, physical or other disabilities (e.g. less visible conditions such as autism or mental health disorders).

‘It [*accommodation of people with disabilities*] is a challenge, because we take it for granted, we work with people in communities, train them to go and reach out into homes and if the material does not cater for those people [*people with disabilities*] then it becomes a challenge, we are not printing anything in Braille, it is a challenge and it is something that we acknowledge needs to change.’ (Manager of NGO focusing on men and boys)

‘I think we could make sure that our staff can interpret in sign language that is very, very important. It will be added value in our programmes, particularly those addressing issues of gender-based violence. The other thing is to utilise more pictures than words, I feel that would add value. Perhaps as part of our induction package we need to make sure that we are able to deliver and make sure that everyone will be able to access our services.’ (Manager from AIDS service organisation)

Representatives from DPOs proposed the development of specific programmes that would address the needs of people with disabilities, including specific programmes around violence. These programmes were envisioned as being comprehensive including a number of areas such as employment, education and violence prevention.

‘Looking at our current programming, it may be ideal to have a specific programme that addresses the needs of [*Deaf*] women and young girls and does not only focus on GBV but focuses on the needs of the women and girls and that includes employability, … economic empowerment, … education, … rights and issues of gender-based violence.’ (Manager of DPO for the Deaf)

Furthermore, enhancing networking among DPOs and NGOs was seen as an opportunity for change in terms of both improving referral to services and gaining support for the organisation.

‘Opportunities that we have, that make us reach out to women and girls with disabilities, are networking with other NGOs or DPOs. We usually refer clients, who we can’t help, to other organisations to assist us. For example we have Lentswe la ba na le Bogole in Francistown and social workers.’ (Manager of AIDS service organisation)

Lastly, participants also used the checklist to identify organisational structures and procedures that their organisation could change internally (Table 3). Most organisations identified adjusting their policies and strategies, and increasing their linkages to poverty alleviation programmes and other NGOs or DPOs as areas that they could improve. Organisations also identified training of staff as opportunities for change, with NGO representatives feeling less confident that they could arrange training related to disability sensitisation, sign language interpretation or screening and identification of disability. Few NGOs and DPOs identified that they could address measures of accessibility of their facilities and services through universal design and reasonable accommodation.

### Needed support from national departments and communities

Participants also identified external structures, procedures and stakeholders that were needed to improve accessibility, inclusion and support and through which their organisations could increase participation and service delivery to women and girls with disabilities. This included areas of national policies and government, increased accessibility of public services, involvement of caregivers, knowledge creation, dissemination and disability inclusive monitoring and evaluation.

Firstly, participants revealed that some of the NGOs and DPOs already benefited from existing policies but that within the mainstream settings policies and services still neglected people with disabilities. In fact, one participant highlighted that the country did not have a specific disability law and that the existing disability policy is old and had been under review for a long time. Hence there was a need to develop policies and regulations.

‘We don’t have any specific law for people with disability. We want to move away from the welfare policy [*the old disability policy*] which is now under review for six years … to people who can participate in issues that affect their lives. So that is the first thing we want to see, policy and law reform, so that we can sign the Convention on the Rights of Persons with Disabilities. But even if we don’t sign our laws should be inclusive to enable people with disabilities to access services and participate in the economic issues, education and so on.’ (Manager of AIDS service organisation)

Furthermore, involvement of people with disabilities and their service organisations in policy design and development was seen as essential to ensure that people with disabilities benefited from them.

‘Government should make policies for us as people with disabilities to benefit from designated programmes and not just come with policies that do not benefit us but are said to be for us.’ (Manager of DPO for people with visual impairments)

Secondly, the inaccessibility of buildings was seen as related to government offices who approve buildings under development; these departments were seen as essential in assuring that buildings were transformed to be accessible to people with disabilities.

‘It [*inaccessibility*] goes back to the council that approves the buildings. When people build they need to undergo a process and submit their plans to the council, who look at the plan and give the go ahead … they also need to take into considerations that there is a certain group [people with disabilities] that also need to be catered for.’ (Manager of AIDS service organisation)

Thirdly, NGOs and DPOs identified the need to train government employees in key services such as the police, justice, health and education to ensure that they understood the need of people with disabilities.

‘Justice still needs a lot of training, our police or anybody who is responsible for justice, like the lawyers who represent the person, they need training.’ (Manager of DPO for people with physical disabilities)

‘The challenges we have are related to language barriers, when something happens to them [*women with disabilities*] … we need to assist them to access judicial services. … If there was someone overseeing disability issues in the northern region especially for the Deaf … that would help.’ (Manager of NGO focusing on violence against women)

Furthermore, caregivers and families were identified as important role players that needed information about care and support for their family members with disabilities. This included the ability to understand abuse and violence and how to prevent it.

‘We talk about abuse even with parents. … Parents of children with disabilities don’t know how to protect them from abuse … parents don’t know what to do. … Parents need to be taught about girls with disabilities so that when your child is not home, they don’t just think that they are playing with others… Social workers should provide parenting lessons on children with disabilities during clinic visits … parents should be grouped and be taught that as people with disabilities we need special care based on our needs.’ (Manager of DPO for people with intellectual disabilities)

Lastly, monitoring and evaluation was seen as an important tool to understand the needs of people with disabilities but also to measure which training and programmes have reached how many people or women with disabilities. Participants here highlighted that tools needed to be developed to monitor the inclusion of people with disabilities.

‘Very important is … that you measure … how many trainings you have that cover these women and girls [*with disabilities*], what kind of training are those and how often have you provided them during a particular period. So it is a matter of developing a tool that will measure the statistics of the women and girls with disabilities.’ (Manager of DPO for all disability types)

## Discussion

The provided sub-study is a first description of the capacity of NGOs and DPOs in Botswana to increase participation of women and girls with disabilities and ensure access to services and programmes addressing violence, including those who are involved in HIV or SRHR programmes. The sub-study is limited to the information provided by the leadership of the NGOs and DPOs participating in this study. None of these organisations had women with disabilities in leadership positions. The fieldwork was led by women with disabilities who prompted these leaders to identify gaps and solutions for their organisations’ activities and services (using the audit and question guide). This approach challenged participants to identify gaps and solutions to increase participation of women and girls with disabilities in their organisations’ activities.

The results show that the leadership of NGOs and DPOs is prepared to drive inclusion of people with disabilities in programmes that address violence and can identify a number of gaps and opportunities.

Firstly, the results suggest that the facilities of NGOs and DPOs lack basic elements of universal design and reasonable accommodation; hence they are inaccessible to some people with disabilities. However, these organisations should provide guidance and good practice to drive inclusion of vulnerable populations such as people with disabilities. Besides potential lack of sufficient funding, lack of awareness and tools to assess accessibility and inclusion contributes to the inaccessibility of NGOs and DPOs. In this study the disability audit enabled participants to identify accessibility and inclusion gaps as well as opportunities for change.

The usage of a disability audits could become a standard tool for NGOs and DPOs and guide the improvement and adaptations of these organisations’ facilities, services and activities. Disability audits for NGOs and DPOs in Africa have not been published, because validated and easy-to-use tools still need to be developed. Hence, the further development and validation of tools such as the one used in this study could become a research priority.

Secondly, the data revealed that NGOs and DPOs had complementary pockets of expertise that could be utilised to improve access to and inclusion in programmes that address violence. On the one hand NGOs had a wide geographical reach and developed expertise to address HIV or violence against women and girls in urban and rural areas. However, NGOs did not include or prioritise disability and their staff lacked knowledge about disability and its intersection with violence. Knowledge gaps included ethical considerations around disability and skills to accommodate disability-related needs. Understanding of disability by NGO representatives was influenced by doctrines common in mainstream and key programmes in the region. For instance, some NGO representatives held the notion that they ‘shouldn’t know the disability status of their clients’. Ethical regulations around confidentiality of somebody’s ‘status’ are common in the context of HIV in Southern Africa, but this approach hinders identification of disability needs and, through this, adjustments that ensure access and equity. Similarly, the generic ‘include all’ approach led to the notion that organisations would include people with disabilities via default. Without understanding of and allocating resource to accommodate disability-related needs this approach is no more than lip service. Representatives from NGOs therefore identified capacity building and programme adaptations as important pillars to mainstream disability across their programmes.

On the other hand, DPOs understood the disability-related needs of their constituency but lacked geographical reach, information on other disability groups and strategies on how to mitigate violence against women and girls with disabilities. As a result, DPOs provided limited support or guidance to NGOs and were not utilised enough to address violence against women and girls with disabilities. This speaks to improvement in networking between NGOs and DPOs, capacity building to increase DPO members’ knowledge on the intersection of disability, gender and violence and the development of strategies to address violence against women and girls with disability. In addition, such training should target NGO and DPO members at the same time so these complementary organisations can learn from each other and build networks for future collaboration.

Thirdly, the study provides the participants’ perspectives on how to increase their organisations’ capacity to promote participation of and reduce violence against women and girls with disabilities. The research tools, such as the audit, and also the participatory approach and leadership of researchers with disabilities, provided an enabling environment in which participants were able to identify their opportunities for change. For instance, participants suggested that NGOs and DPOs needed to develop their organisations’ polices and plans to prioritise disability-related needs and violence prevention and through this allocate resources to enhance accessibility, inclusion and focus on prevention of violence against women and girls with disabilities. Participants also highlighted the need to employ women with disabilities and to conduct training of staff to address disability needs in the context of existing programmes that aim to prevent violence or address HIV or other SRHR issues. These suggestions blend in with disability mainstreaming approaches practised in other countries in Africa (Christopher Blind Mission & Comprehensive Community Based Rehabilitation Tanzania 2012). These approaches promote adjustments of mainstream services in terms of increased accessibility and inclusion of people with disabilities both as clients or patients and staff.

Participants suggested also including disability-focused approaches such as the training of staff on the intersection of disability and violence and the development of focused programmes to address violence against women and girls with disabilities. In southern and eastern Africa disability-focused approaches are promoted to address specific gaps or vulnerabilities. In the context of violence against women and girls with disabilities this has specifically been discussed in terms of communication support for police and judicial services and accessibility of comprehensive sexuality education (UNFPA 2018a, 2018b).

The combination of the disability-mainstreaming and disability-focused approaches is internationally known as the twin-track approach, which has been promoted in developmental work with people with disabilities (Christopher Blind Mission & Comprehensive Community Based Rehabilitation Tanzania 2012; Handicap International 2014). The twin-track approach may therefore also be suitable for NGOs and DPOs to drive inclusion and accessibility in programmes addressing violence in Botswana.

Lastly, the ALIGHT study has shown that DPOs and NGOs operate in an environment where both national policies and public services are not addressing disability-related needs adequately (Hanass-Hancock et al. 2018a, 2018b). Inaccessibility and lack of service delivery increase the costs that occur when DPOs and NGO work with people with disabilities, as individuals or organisations have to provide the resources to accommodate disability-related needs (e.g. transport, sign language) (Banks & Polack 2013; Hanass-Hancock et al. 2017). Participants, therefore, highlighted that policymakers and staff at public services need disability inclusion training, that caregivers need sensitisation and support and that facilities need to be adapted to be accessible.

Participants emphasised the need to develop national disability regulations, policies and laws that address the lack of disability inclusion in service delivery and drive training of staff and resource allocations. This echoes the findings of the ALIGHT situation analysis, which showed that Botswana needs to develop disability policies and laws and that disability inclusion and accessibility need to be mainstreamed across HIV, GBV and SRHR policies and strategies (Hanass-Hancock et al. 2018b). During the drafting of this article, Botswana was developing a new disability policy and strategy and has indicated that the country will sign the CRPD. Hence, there is ample opportunity to ensure that the prevention of violence against women and girls with disabilities is integrated into the new national disability policy and law. Policy and programme reform can formalise disability mainstreaming and drive specialised projects that address vulnerability of women and girls with disabilities (Christopher Blind Mission & Comprehensive Community Based Rehabilitation Tanzania 2012; Handicap International 2014; UNAIDS 2017). Such a process will also enable resource allocations and the development of monitoring and evaluation systems that can track progress in disability inclusion, increase in participation and reduction in violence against women and girls with disabilities (Hanass-Hancock et al. 2018b). For instance, existing programmes and national surveys such as the GBV indicator survey of the General Household Survey can include disability indicators and through this inform programmes that address violence against women. Existing NGO programmes that address violence against women can include disability questions in their reporting and evaluation processes and DPOs can measure provision of disability training and support to these NGOs. The important denominator for all these reforms has been highlighted by the participants and lies within building the capacity of service providers, policymakers and NGO and DPO staff to better understand and address the increased vulnerability of women and girls with disabilities to violence. Enabling the participation and leadership of women with disabilities in this context is a necessity.
